# Optimization of Aspiration Pressure in Oocyte Retrieval Using Reduced Needles

**DOI:** 10.7759/cureus.92106

**Published:** 2025-09-11

**Authors:** Akira Nakabayashi, Shuko Murata, Yu Horibe, Tomomi Hashimoto, Tomoko Goto, Tsutomu Tabata

**Affiliations:** 1 Department of Obstetrics and Gynecology, Tokyo Women's Medical University, Tokyo, JPN

**Keywords:** aspiration pressure, fertilization rate, in vitro fertilization (ivf), oocyte retrieval, reduced needle

## Abstract

Introduction: In recent years, reduced needles have been developed with a thin tip and thick shaft, offering reduced pain compared to conventional thick needles without compromising clinical outcomes, leading to increased utilization. However, there has been no investigation of optimal aspiration pressure for reduced needles. Generally, aspiration pressure needs to be increased when the needle diameter is reduced. In this study, we investigated the optimal pressure for reduced needles with two different diameters.

Methods: We conducted a retrospective analysis of 223 cycles of oocyte retrieval performed at our facility from January 2023 to June 2025. Reduced needles with a 20-gauge tip and 17-gauge shaft were used, comparing aspiration pressures of 230 mmHg and 160 mmHg. We compared age, anti-Müllerian hormone (AMH) levels, procedure time, number of punctured follicles, number of retrieved oocytes, and number of fertilized oocytes between the two pressure groups.

Results: No significant differences were observed between the two groups in age, AMH levels, number of punctured follicles, number of retrieved oocytes, or number of fertilized oocytes. However, procedure time was significantly shorter in the 160 mmHg group when AMH ≥1.2 (p=0.009). While no significant difference was found in oocyte retrieval rates, fertilization rates were significantly higher in the 160 mmHg group for both AMH ≥1.2 and AMH <1.2 cases (p=0.009, 0.001, respectively).

Conclusions: In reduced needles, high aspiration pressure was associated with decreased fertilization rates in our study. Our findings suggest that adjusting aspiration pressure according to the thick shaft diameter rather than the thin tip diameter may be preferable.

## Introduction

Transvaginal ultrasound-guided oocyte retrieval was first reported in 1985 and has now become a fundamental technique [1]. The transvaginal approach for oocyte collection reduced invasiveness, leading to the use of local anesthesia or sedation instead of general anesthesia [2]. Needles used for oocyte retrieval range from 17-gauge to 21-gauge. Thicker needles are less prone to bending, and needle tips are less likely to deviate from the puncture guide, but they cause greater pain and increased bleeding. Conversely, thinner needles are more prone to bending and carry the risk of needle tip deviation from the puncture guide, potentially losing visualization on the screen, but they cause less pain and allow faster post-retrieval recovery. Although prospective studies on oocyte retrieval needles are limited, it has been reported that reducing needle diameter from 15-gauge to 17/18-gauge decreased pain without affecting the number of retrieved oocytes, oocyte quality, or pregnancy rates [3-6]. In prospective research on further needle diameter reduction, Kushnir et al. reported that 20-gauge needles, compared to 17-gauge needles, did not affect the number of retrieved oocytes but prolonged procedure time [3].

Recently, puncture needles with reduced diameter only at the tip that penetrates tissue (reduced needles) have been developed [7]. The thin tip reduces bleeding and pain, while the thick shaft maintains needle rigidity, preventing needle bending and shortening procedure time. In a randomized controlled trial (RCT) comparing conventional thick needles with reduced needles having only a thin tip, Wikland et al. reported that using 20/17-gauge reduced needles compared to 17-gauge puncture needles showed no differences in procedure time, oocyte retrieval rate, fertilization rate, number of frozen embryos, or pregnancy rate, while pain and genital bleeding were reduced [7]. Additionally, Buisman et al. reported that 20/17-gauge reduced needles, compared to 16-gauge puncture needles, showed equivalent oocyte retrieval outcomes with reduced pain during retrieval and up to two days post-retrieval [8].

We previously compared 20/17-gauge reduced needles with 20-gauge needles and reported that reduced needles shortened procedure time and reduced anesthetic usage, but decreased fertilization rates [9]. During this comparison, we set the aspiration pressure at 230 mmHg to match the 20-gauge tip, whereas Wikland et al. and Buisman et al., who both compared reduced needles with thick needles (17-gauge and 16-gauge, respectively), used 90-120 mmHg and 120 mmHg, respectively. The relationship between needle diameter and aspiration pressure can be explained by the Hagen-Poiseuille law, where flow rate is proportional to the fourth power of the needle radius, and pressure loss is inversely proportional to the square of the needle diameter. Generally, thinner needles require higher aspiration pressure settings, but reduced needles have both thick and thin diameter sections, and no studies have examined aspiration pressure for reduced needles. The objective of this retrospective study was to determine the optimal aspiration pressure for reduced oocyte retrieval needles (20-gauge tip; 17-gauge shaft) by comparing clinical outcomes between two different aspiration pressure settings. The primary outcomes were fertilization rates and procedure time; secondary outcomes included oocyte retrieval rate and number of retrieved oocytes.

## Materials and methods

Of 229 cycles of oocyte retrieval performed at our facility from January 2023 to June 2025, we analyzed 223 cycles after excluding four cycles of oocyte cryopreservation and two cycles where intracytoplasmic sperm injection (ICSI) could not be performed due to poor semen parameters. Information regarding oocyte retrieval was extracted from medical records. The extracted parameters included age, anti-Müllerian hormone (AMH) levels, oocyte retrieval start time, oocyte retrieval end time, number of punctured follicles, number of retrieved oocytes, number of fertilized oocytes, and aspiration pressure. This study was approved by the university ethics committee under the comprehensive research protocol "Retrospective Study on Treatment Outcomes in Assisted Reproductive Technology" (approval number 5704). This approval covers multiple related studies aimed at improving IVF outcomes, including our previous publication and the current investigation on aspiration pressure optimization [9].

For comparative analysis, patients were classified into two groups: those with expected normal ovarian response (AMH ≥1.2) and those with expected poor ovarian response (POR) (AMH <1.2). Within each group, we compared outcomes between two different aspiration pressures for the following parameters: age, AMH level, procedure time, number of punctured follicles, number of retrieved oocytes, number of fertilized oocytes, oocyte retrieval rate, and fertilization rate. Procedure time was defined as the duration from the start of vaginal irrigation to the completion of hemostasis confirmation and speculum removal after puncture. The oocyte retrieval rate was calculated by dividing the total number of retrieved oocytes by the total number of punctured follicles, and the fertilization rate was calculated by dividing the total number of fertilized oocytes by the total number of retrieved oocytes. For comparison between the two groups, the Mann-Whitney U-test for continuous variables and Fisher's exact test were used for dichotomous variables. All significance tests were two-sided and conducted at the 0.05 significance level. All statistical analyses were performed using statistical software, JMP Pro version 17 (SAS Institute Inc., Cary, NC, USA).

Oocyte retrieval at our facility is primarily performed by two reproductive medicine specialists using standardized procedures. According to our protocol, only follicles with a diameter of 10 mm or greater were punctured for aspiration. We used Vitrolife AB (Sweden) puncture needles with a total length of 35 cm, consisting of a 5 cm tip with 20-gauge diameter (outer diameter 0.9 mm) and a 30 cm shaft with 17-gauge diameter (outer diameter 1.4 mm). For follicle puncture aspiration pressure, we used the Cook Aspiration Unit (Cook Medical) set at either 230 mmHg or 160 mmHg. For sedation, 200 mg of thiopental sodium was administered intravenously as a bolus, with additional doses given as needed based on patient pain levels.

For patients without diminished ovarian reserve, controlled ovarian hyperstimulation was basically performed; for patients with diminished ovarian reserve, mild ovarian stimulation was performed. Ovarian stimulation was performed using follicle-stimulating hormone (urinary or recombinant) or human menopausal gonadotropin in combination with GnRH agonist or antagonist or progestin according to each patient's status. Dosage was also set individually, taking into account age, AMH level, body weight, and previous oocyte retrieval outcomes. The timing of oocyte retrieval was determined based on follicle development and estradiol levels. Human chorionic gonadotropin injection was administered 34 hours prior to oocyte retrieval. The decision to use IVF or ICSI was based on sperm status and previous treatment history. Fertilization was assessed by confirming the presence of two pronuclei on the morning following oocyte retrieval, approximately 18 hours after insemination or ICSI.

## Results

Aspiration pressure was set at 230 mmHg from January 2023 to April 2024, and at 160 mmHg from May 2024 to June 2025. There were 113 cycles in the 230 mmHg group and 110 cycles in the 160 mmHg group. Among these, 111 cycles had AMH ≥1.2, with 51 cycles in the 230 mmHg group and 60 cycles in the 160 mmHg group. Conversely, 112 cycles had AMH <1.2, with 62 cycles in the 230 mmHg group and 50 cycles in the 160 mmHg group.

Table 1 shows the results of per-retrieval cycle analysis. Regarding patient background characteristics of age and AMH levels, no significant differences were observed between the 230 mmHg and 160 mmHg groups. For the number of punctured follicles, number of retrieved oocytes, and number of fertilized oocytes, no significant differences were found between the 230 mmHg and 160 mmHg groups in both AMH ≥1.2 and AMH <1.2 subgroups. Regarding procedure time, no significant difference was observed in the AMH <1.2 group (p=0.11), but in the AMH ≥1.2 group, the procedure time was 11.0±4.1 minutes in the 230 mmHg group and 9.4±4.1 minutes in the 160 mmHg group, showing a significant reduction in the lower pressure 160 mmHg group (p=0.009).

**Table 1:** Baseline characteristics and oocyte retrieval procedure data in each group AMH: anti-Mullerian hormone

	AMH ≥1.2			AMH <1.2		
	230 mmHg	160 mmHg	P-value	230 mmHg	160 mmHg	P-value
Cycles (n)	51	60		62	50	
Age (years)	36.1±4.8	35.1±4.7	0.29	41.2±3.8	39.8±4.6	0.09
AMH (ng/mL)	4.1±2.7	4.2±3.1	0.77	0.49±0.25	0.51±0.30	0.75
Procedure time (min)	11.0±4.1	9.4±4.1	0.009	6.3±2.8	5.5±2.5	0.11
Follicles punctured (n)	21.3±14.7	17.4±12.0	0.19	4.5±2.8	4.5±4.0	0.33
Oocytes retrieved (n)	14.2±10.0	12.2±8.0	0.51	2.8±2.5	3.2±3.5	0.84
Oocytes fertilized (n)	8.0±6.6	7.8±6.6	0.96	1.5±1.5	2.3±2.7	0.42

Table 2 shows the results of per-follicle and per-oocyte analysis. Oocyte retrieval rates showed no significant differences in either group: for AMH ≥1.2, rates were 66.4% (722/1087) and 70.0% (731/1044) in the 230 mmHg and 160 mmHg groups, respectively; for AMH <1.2, rates were 63.1% (176/279) and 70.4% (159/226) in the 230 mmHg and 160 mmHg groups, respectively (p=0.09, 0.08). However, fertilization rates showed significant differences in both groups: for AMH ≥1.2, rates were 56.7% (409/722) and 63.9% (467/731) in the 230 mmHg and 160 mmHg groups, respectively; for AMH <1.2, rates were 53.4% (94/176) and 71.1% (113/159) in the 230 mmHg and 160 mmHg groups, respectively (p=0.005, 0.001).

**Table 2:** Oocyte retrieval rates and fertilization rates AMH: anti-Mullerian hormone

	AMH ≥1.2			AMH <1.2			
	230 mmHg	160 mmHg	P-value	230 mmHg	160 mmHg	P-value	
Oocyte retrieval rate (%)	66.4	70.0	0.08	63.1	70.4	0.09	
Total follicles punctured (n)	1087	1044		279	226		
Total oocytes retrieved (n)	722	731		176	159		
Fertilization rate (%)	56.7	63.9	0.005	53.4	71.1	0.001	
Total oocytes retrieved (n)	722	731		176	159		
Total oocytes fertilized (n)	409	467		94	113		

## Discussion

Although higher aspiration pressure theoretically increases flow rate and would be expected to shorten procedure time, in practice, the lower pressure of 160 mmHg resulted in shorter procedure times in the AMH ≥1.2 group. Although not statistically significant, there was a trend toward fewer punctured follicles in the 160 mmHg group, which may have influenced procedure time. Based on our data, it appears that above a certain threshold aspiration pressure, lower pressure does not prolong procedure time. Regarding oocyte retrieval rates, no significant differences were observed between aspiration pressures. However, concerning fertilization rates, considering that typical fertilization rates are approximately 67% for IVF and 70% for ICSI, the fertilization rates with 230 mmHg (56.7% for AMH ≥1.2 and 53.4% for AMH <1.2) were low, suggesting that excessively high pressure adversely affects fertilization rates [10].

We decided to reduce aspiration pressure based on the observed decrease in fertilization rates when we changed from 20-gauge needles to 20/17-gauge reduced needles [9]. Fertilization rates decreased from 66.8% to 56.8% for AMH ≥1.2 and from 63.5% to 55.7% for AMH <1.2 [9]. Since there were no changes in oocyte retrieval methods or culture techniques, we hypothesized that aspiration pressure was the influencing factor and reduced the pressure from 230 mmHg to 160 mmHg to compare outcomes. The 230 mmHg pressure was the recommended aspiration pressure for 20-gauge needles, and we had set the same pressure when using 20/17-gauge reduced needles. By reducing the aspiration pressure, fertilization rates improved to 63.9% for AMH ≥1.2 and 71.1% for AMH <1.2, reaching levels that met the Vienna consensus competency values for fertilization rates (IVF: 60%, ICSI: 65%) [10]. Follicle diameter is a factor that affects fertilization rates, and Wirleitner et al. reported that small follicles of 8-12 mm had lower proportions of mature (M2) oocytes and decreased fertilization rates compared to medium-sized follicles of 13-24 mm or large follicles over 24 mm [11]. Since our facility punctures small follicles of approximately 10 mm, we believe this contributes to our tendency toward lower fertilization rates. It should be noted that higher fertilization rates correlate with higher cumulative live birth rates, making fertilization rate comparison useful when evaluating oocyte retrieval outcomes [12].

No consensus has been reached regarding optimal aspiration pressure, with most facilities setting 100-200 mmHg for 17-18 gauge puncture needles [13]. Reports on aspiration pressure are also limited. Kim et al. compared two aspiration pressures of 120 mmHg and 150 mmHg, reporting no difference in live birth rates but higher oocyte and embryo yields with 150 mmHg [14]. Kumaran et al. reported that in low antral follicle count (AFC) patients using 17-gauge needles, 140 mmHg aspiration pressure resulted in higher oocyte yield and pregnancy outcomes compared to 120 mmHg flushing and aspiration [15]. Although conducted in pigs, studies have shown that higher aspiration pressure reduces oocyte retrieval rates and increases the number of oocytes with stripped granulosa cells at high pressures [16]. High aspiration pressure may cause follicle collapse and oocyte damage due to shear forces and turbulence at the needle tip, while low aspiration pressure may result in insufficient follicular fluid aspiration. In this study, we used reduced needles and set the pressure at 160 mmHg, which was 10-20 mmHg higher than previous reports due to the thinner tip. No previous comparisons have been made with reduced needles, and our comparison suggests that aspiration pressure should be set according to the thick shaft rather than the thin tip.

The reason for stratifying patients into AMH ≥1.2 and AMH <1.2 groups was the significant difference in the number of punctured follicles: an average of 19.2 follicles in the AMH ≥1.2 group versus 4.5 follicles in the AMH <1.2 group. The cutoff value of 1.2 is used in the POSEIDON criteria for predicting POR. The high proportion of patients with AMH <1.2 reflects the characteristics of infertility treatment in Japan, where the target age is high, attributed to delayed marriage and the fact that oocyte donation is not commonly practiced [17-19]. The target ages in reports on reduced needle outcomes by Wikland and Buisman were 34 and 33 years, respectively, which approximate our AMH <1.2 group, suggesting that grouping by ovarian reserve is clinically useful.

One limitation of this study is its retrospective design with temporal allocation by time period. Although the same physicians and embryologists performed procedures throughout the 2.5-year study period with no major protocol changes, suggesting minimal bias, the sequential nature of group allocation cannot completely exclude the possibility of temporal confounding factors. A second limitation relates to subject selection: this study included all treatment cycles when the same patient underwent multiple treatments. While this is unlikely to significantly impact our primary objective of comparing aspiration pressures, focusing on first treatment cycles only would be preferable for accurate calculation of oocyte retrieval and fertilization rates. A third limitation concerns outcome evaluation parameters. Ideally, oocyte retrieval outcomes should include pregnancy rates, but these are significantly influenced by causes of infertility, indications for IVF, and gamete status, so pregnancy rates were not included as evaluation parameters in this study.

## Conclusions

In oocyte retrieval using reduced needles, different aspiration pressures showed no differences in procedure time or oocyte retrieval rates but did show differences in fertilization rates. In this study, higher aspiration pressure (230 mmHg) was associated with decreased fertilization rates compared to lower pressure (160 mmHg). Our findings suggest that pressure settings may be more appropriately based on the thick shaft diameter rather than the thin tip diameter. Using 160 mmHg achieved fertilization rates equivalent to Vienna consensus competency values without prolonging procedure time. However, these results are based on comparison of only two pressure settings with one needle design, and validation through larger multicenter studies would strengthen these preliminary findings.

## References

[1] Wikland M, Enk L, Hamberger L (1985). Transvesical and transvaginal approaches for the aspiration of follicles by use of ultrasound. Ann N Y Acad Sci.

[2] Kwan I, Bhattacharya S, Knox F, McNeil A (2006). Conscious sedation and analgesia for oocyte retrieval during IVF procedures: a Cochrane review. Hum Reprod.

[3] Kushnir VA, Kim A, Gleicher N, Barad DH (2013). A pilot trial of large versus small diameter needles for oocyte retrieval. Reprod Biol Endocrinol.

[4] Aziz N, Biljan MM, Taylor CT, Manasse PR, Kingsland CR (1993). Effect of aspirating needle calibre on outcome of in-vitro fertilization. Hum Reprod.

[5] Awonuga A, Waterstone J, Oyesanya O, Curson R, Nargund G, Parsons J (1996). A prospective randomized study comparing needles of different diameters for transvaginal ultrasound-directed follicle aspiration. Fertil Steril.

[6] Nakagawa K, Nishi Y, Kaneyama M, Sugiyama R, Motoyama H, Sugiyama R (2015). The effect of a newly designed needle on the pain and bleeding of patients during oocyte retrieval of a single follicle. J Reprod Infertil.

[7] Wikland M, Blad S, Bungum L, Hillensjö T, Karlström PO, Nilsson S (2011). A randomized controlled study comparing pain experience between a newly designed needle with a thin tip and a standard needle for oocyte aspiration. Hum Reprod.

[8] Iduna Antigoni Buisman ET, de Bruin JP, Maria Braat DD, van der Steeg JW (2021). Effect of needle diameter on pain during oocyte retrieval-a randomized controlled trial. Fertil Steril.

[9] Nakabayashi A, Hashimoto Y, Horibe Y, Hashimoto T, Murata S, Kakogawa J (2024). Comparison of a reduced needle with a thin tip and a standard thin needle (same width overall) for oocyte retrieval. Cureus.

[10] ESHRE Special Interest Group of Embryology and Alpha Scientists in Reproductive Medicine (2017). The Vienna consensus: report of an expert meeting on the development of ART laboratory performance indicators. Reprod Biomed Online.

[11] Wirleitner B, Okhowat J, Vištejnová L (2018). Relationship between follicular volume and oocyte competence, blastocyst development and live-birth rate: optimal follicle size for oocyte retrieval. Ultrasound Obstet Gynecol.

[12] Scaravelli G, Zacà C, Levi Setti PE (2021). Fertilization rate as a novel indicator for cumulative live birth rate: a multicenter retrospective cohort study of 9,394 complete in vitro fertilization cycles. Fertil Steril.

[13] D'Angelo A, Panayotidis C, Amso N (2019). Recommendations for good practice in ultrasound: oocyte pick up(†). Hum Reprod Open.

[14] Kim SW, Kim HY, Han JY, Kim H, Ku SY (2025). Optimal aspiration pressure of suction pump for oocyte retrieval in infertile patients undergoing in vitro fertilization. PLoS One.

[15] Kumaran A, Narayan PK, Pai PJ, Ramachandran A, Mathews B, Adiga SK (2015). Oocyte retrieval at 140-mmHg negative aspiration pressure: a promising alternative to flushing and aspiration in assisted reproduction in women with low ovarian reserve. J Hum Reprod Sci.

[16] Brüssow KP, Torner H, Rátky J, Hunter MG, Nürnberg G (1997). Ovum pick up in swine: the influence of aspiration vacuum pressure on oocyte recovery from preovulatory follicles. Acta Vet Hung.

[17] Katagiri Y, Jwa SC, Kuwahara A (2023). Assisted reproductive technology in Japan: a summary report for 2020 by the Ethics Committee of the Japan Society of Obstetrics and Gynecology. Reprod Med Biol.

[18] Sugino N (2018). First healthy baby by anonymous oocyte donation in Japan. Reprod Med Biol.

[19] Yamamoto N, Hirata T, Izumi G (2018). A survey of public attitudes towards third-party reproduction in Japan in 2014. PLoS One.

